# Effects of Reallocating Time Spent in Different Physical Activity Intensities on Sarcopenia Risk in Older Adults: An Isotemporal Substitution Analysis

**DOI:** 10.3390/biology11010111

**Published:** 2022-01-10

**Authors:** Jort Veen, Diego Montiel-Rojas, Fawzi Kadi, Andreas Nilsson

**Affiliations:** School of Health Sciences, Örebro University, 70182 Örebro, Sweden; jort.veen@oru.se (J.V.); diego.montiel@oru.se (D.M.-R.); fawzi.kadi@oru.se (F.K.)

**Keywords:** aging, muscle strength, muscle mass, exercise

## Abstract

**Simple Summary:**

The role of daily time spent sedentary and in different intensities of physical activity (PA) for maintenance of muscle health is currently unclear. Therefore, we investigated the impact of reallocating time spent in different PA intensities on a sarcopenia risk score (SRS) in older adults, while considering PA type (muscle strengthening activities, MSA) and protein intake. In the present study, we show for the first time that reallocating sedentary time with at least light-intensity PA was significantly related to a lower SRS, which remained evident after adjustment by PA type (MSA) and protein intake. Similarly, reallocating time in light- to moderate-to-vigorous-intensity PA was related to a significantly lower SRS. Our results emphasize the importance of displacing sedentary behaviours for more active pursuits, where PA of even light intensities may alleviate age-related deteriorations of muscle health in older adults.

**Abstract:**

The role of daily time spent sedentary and in different intensities of physical activity (PA) for the maintenance of muscle health currently remains unclear. Therefore, we investigated the impact of reallocating time spent in different PA intensities on sarcopenia risk in older adults, while considering PA type (muscle strengthening activities, MSA) and protein intake. In a sample of 235 community-dwelling older adults (65–70 years), a sarcopenia risk score (SRS) was created based on muscle mass assessed by bioimpedance, together with handgrip strength and performance on the five times sit-to-stand (5-STS) test assessed by standardized procedures. Time spent in light-intensity PA (LPA), moderate-to-vigorous PA (MVPA), and being sedentary was assessed by accelerometry, and PA type (MSA) by self-report. Linear regression models based on isotemporal substitution were employed. Reallocating sedentary time to at least LPA was significantly (*p* < 0.05) related to a lower SRS, which remained evident after adjustment by PA type (MSA) and protein intake. Similarly, reallocating time in LPA by MVPA was related to a significantly (*p* < 0.05) lower SRS. Our results emphasize the importance of displacing sedentary behaviours for more active pursuits, where PA of even light intensities may alleviate age-related deteriorations of muscle health in older adults.

## 1. Introduction

The ageing process is accompanied by a progressive deterioration of muscle health, including a decline in muscle mass, strength and physical performance. This age-related deterioration of muscle health increases the risk of developing sarcopenia, a condition defined as a progressive and generalized skeletal muscle disorder characterized by an increased risk of falls, physical disability and mortality (European Working Group on Sarcopenia in Older People (EWGSOP)) [1].

In general, the beneficial role of physical activity (PA) in the preservation of muscle health is well documented. While prolonged periods of sedentary behaviour have been shown to contribute to an increased sarcopenia risk in older adults [2,3], time spent in moderate-to-vigorous PA (MVPA) has been shown to be positively related to muscle mass, muscle strength, and physical performance [4,5,6,7,8,9]. However, uncertainties remain about the role of light-intensity PA (LPA) in the preservation of muscle health, which is unfortunate given that older adults are likely to spend more time in LPA at the expense of MVPA. Indeed, while one report indicated a beneficial impact of LPA on indicators of muscle health [8], others did not [5,7].

Interestingly, while most previous studies have addressed the influence of separate dimensions of PA behaviours (e.g., time in LPA and MVPA) on muscle health [4,5,6,7,8,9,10], the fact that daily time spent in one PA intensity inevitably displaces time spent in another has not always been considered. In this respect, isotemporal substitution modelling based on regression analysis [11] has been employed in order to estimate the effect on various health outcomes associated with hypothetical time reallocation of different PA intensities [10,12,13,14,15,16]. There is currently a paucity of studies taking advantage of the isotemporal modelling in order to examine the impact of PA time patterns on muscle health in older adults. Recent studies reported that replacement of a given period of sedentary time with MVPA, but not LPA, was associated with lower sarcopenia risk [17,18]. In contrast, a recent study failed to demonstrate any effect of reallocating time spent in sedentary by LPA or MVPA on fat-free mass [19]. In addition, alongside PA intensity time patterns, activity type (endurance vs. strength) may yield different effects on muscle health, where muscle strengthening activities (MSA) are generally regarded as an efficient strategy for promotion of improvements in muscle mass and strength [20], and engagement in MSA has been shown to reduce sarcopenia risk [21,22,23,24,25]. Furthermore, the confounding influence of dietary protein intake, a well-established driver for preservation of muscle health [26,27], needs to be accounted for, when depicting the links between distribution of PA intensity time patterns and indicators of muscle health in older adults.

Therefore, the aim of the present study was to determine the impact of reallocating time in different PA intensities on indicators of muscle mass and function in older adults, while considering physical activity type (MSA) and adherence to guidelines for protein intake.

## 2. Materials and Methods

### 2.1. Participants

Two-hundred and fifty-two older men and women (65–70 years) were recruited through a local advertisement. Participants were included based on absence of overt disease, diabetes, cardiovascular and psychiatric conditions and disabilities that limit mobility. All investigations were conducted according to the principles set by the Declaration of Helsinki and participants were provided oral and written information about the study and informed consent was obtained. The study protocol was approved by the Swedish Ethical Review Authority, Sweden (2017/511).

### 2.2. Anthropometrics

Body weight and height were measured using standard procedures. Waist circumference (WC) was measured to the nearest 0.1 cm at the midpoint between the iliac crest and lower costal margin. Skeletal muscle mass was determined after an overnight fast based on bioelectrical impedance analysis (Tanita MC-780, Tanita Amsterdam, The Netherlands) and using the equation of Janssen et al. [28].

### 2.3. Assessment of Physical Activity Behaviours

PA was assessed using the Actigraph GT3x (Actigraph, Pensacola, FL, USA) accelerometer during a week as previously described [12]. Wear time for a minimum of four days with at least ten hours per day was required for inclusion. The following accelerometer count cut-points (counts per minute; CPM) were used: >100 CPM for sedentary time (SED) (e.g., TV viewing), >100–2019 for LPA (e.g., slow walking), and >2019 CPM for MVPA (e.g., brisk walking) [29]. MSA was assessed using the EPAQ2 questionnaire [30], where participants reported on frequency of MSA including activities such as strength training and gym-based exercises during the last 12 months. Based on reported frequencies, participants were stratified based on whether they reported MSA once a week (yes/no).

### 2.4. Assessment of Protein Intake

Dietary protein intake was determined using a 90-item food-frequency questionnaire (FFQ) as previously described [24]. In brief, all participants were instructed (face-to-face) on how to fill out the FFQ. The FFQ has 9 fixed alternatives as follows: never, occasionally, 1–3 times/month, 1 time/week, 2–3 times/week, 4–6 times/week, 1 time/day, 2–3 times/day, ≥4 times/day.

### 2.5. Assessment of Indictors of Sarcopenia Risk

Handgrip strength (HG) was assessed by standardized procedures using a Jamar handheld dynamometer (Patterson Medical, Warrenville, IL, USA). A five times sit-to-stand test (5-STS) was performed, whereby participants were instructed to start from a standing upright position and to sit down in a chair and repeat this sequence 5 times. Skeletal muscle mass was then normalized by body weight to obtain the skeletal muscle mass index (SMI, kg/BW). A continuous sarcopenia risk score (SRS) was created based on skeletal muscle index (SMI), HG and 5-STS, which represent indicators of muscle strength and quantity in accordance with the recent operational definition of sarcopenia [1]. Each component of SRS was first standardized (z-scores) in men and women separately and then averaged into one gender-combined composite risk score (SRS) as previously described [27], where a high SRS denotes a higher sarcopenia risk.

### 2.6. Statistical Analyses

Data are presented as mean and standard deviation unless otherwise stated. Differences between males and females were determined by either independent sample t-tests or chi-square tests. First, simple linear regression analysis was used to explore associations between time spent in different PA intensities modelled in 10 min periods and SRS and its subcomponents. Due to differences in original units, all dependent variables were standardized prior to analysis in order to retrieve comparable effect outcomes. Second, isotemporal substitution modelling was employed to investigate the effect on indicators of muscle health by hypothetical changes in time spent in different PA intensities, while holding total wear time constant. All models were adjusted by waist circumference (WC) based on established metabolic risk cut-points of ≥94 cm for men and ≥80 cm for women [30], adherence (yes/no) to recommendations on protein intake (1.1 g·kg^−1^ BW) (Model 1) and additionally adjusted by type of PA (MSA, yes/no) (Model 2). Given there were no significant impacts on SRS by age, medication use or tobacco use, these variables were omitted in the final models to retain statistical power. Assumptions behind multiple regression analysis, including linearity and collinearity, were fulfilled. A priori power calculation, conducted by G-Power statistical software, showed that small to moderate effect sizes are detected with a power of ≥80% when based on our sample size and an alpha level set to 0.05. All analyses were conducted using SPSS version 27.

## 3. Results

For the present study, 235 community-dwelling older men (*n* = 88) and women (*n* = 147) with complete data were included. The general characteristics of the studied population are presented in Table 1. Older men spent significantly more time in SED (*p* < 0.05) and significantly less time in LPA (*p* < 0.05) compared to older women, with no differences in MVPA time (Table 1). Additionally, older men had significantly higher SMI and HG compared to women (*p* < 0.05) with no differences in 5-STS (Table 1). Current medication and tobacco use as well as past tobacco use were 45%, 8% and 55%, respectively.

We first investigated associations between time spent sedentary and in different physical activity intensities and SRS and its components (Table 2). More time spent sedentary was significantly associated with higher sarcopenia risk and lower SMI, HG and 5-STS (*p* < 0.05). More time spent in MVPA was significantly associated with lower SRS and higher SMI, HG and 5-STS (*p* < 0.05). Finally, more time spent in LPA was significantly associated with a lower SRS and higher SMI (*p* < 0.05).

Furthermore, we sought to investigate the impact of reallocating time in different PA intensities on indicators of sarcopenia risk while adjusting for wearing time, WC, and protein intake (Table 3). The results showed that replacement of time in SED with at least LPA was associated with a significantly (*p* < 0.05) lower SRS (model 1). Further replacement of time in LPA with MVPA was associated with a significantly (*p* < 0.05) lower SRS. Importantly, further adjustment by activity type (MSA) left the model outcomes unchanged (model 2).

Analysis of single components of SRS showed that replacement of time in SED by MVPA was associated with a significantly (*p* < 0.05) higher SMI, HG and 5-STS performance (Table 3). Similarly, displacing time in LPA by MVPA was associated with a significantly (*p* < 0.05) higher SMI and HG, while no impact on 5-STS was observed. Interestingly, displacement of time in SED by LPA was related to a significantly (*p* < 0.05) higher SMI, with no corresponding impacts on HG and 5-STS. Finally, adjustment by activity type (MSA) did not alter the above associations (model 2).

## 4. Discussion

A novel finding of the present study was that replacement of a 10 min bout of daily sedentary time with a corresponding bout of at least LPA is associated with a lower sarcopenia risk in older adults, with greater benefits on SRS and its components when further displacing time bouts of LPA by corresponding bouts of MVPA. Importantly, the effects of time reallocation in different intensities were not influenced by the type of activities performed, which highlights the importance of displacing sedentary behaviours for a variety of more physically active pursuits for maintenance of muscle health in older adults.

Our study indicates that displacement of sedentary time for an equivalent time bout in LPA was associated with a significantly lower sarcopenia risk score in older adults. In line with this finding, a recent study based on a Japanese population of older adults showed that replacement of sedentary time with LPA was associated with a higher 5-STS performance, and thus lower sarcopenia risk, although odds ratios for having sarcopenia were not significantly reduced [18]. In contrast, there were no beneficial impacts of LPA on components of sarcopenia risk in a Spanish cohort of older adults [17] (the Toledo Study of Healthy Aging, TSHA–2019). Of note, participants in TSHA were on average 78 years old and were reported to accumulate a daily average of 60 min of MVPA, with the subsample of sarcopenic adults averaging 45 min/day of MVPA, which is unexpected as it is above the average amount accumulated by our sample of apparently healthy older adults and equivalent to twice the recommended amount of weekly MVPA time [31]. Therefore, the fact that replacement of sedentary time with time in LPA was beneficially linked to SRS in ours, but not in the study by Sanchez-Sanchez [17], is likely due to the use of different definitions of MVPA, with a considerably lower intensity threshold used in the latter study. Nevertheless, our finding supports previous data indicating that PA intensities below the MVPA threshold may infer beneficial health effects, including reduced risk of sarcopenic obesity, reduced adiposity, blood pressure, markers of lipid and glucose metabolism, and mortality [8,32,33,34].

Interestingly, replacing a given time bout of LPA with MVPA, while keeping daily sedentary time constant, was further associated with a significantly lower sarcopenia risk in our sample of older men and women. In accordance with our finding, a significant reduction in likelihood of having sarcopenia was observed when displacing LPA with MVPA in a Spanish cohort of older adults [17]. Altogether, our findings support global guidelines for health enhancing physical activity, targeting daily accumulation of time in MVPA, and further emphasizes the benefit of limiting daily sedentary time and increase time in activities of any intensity, including light intensity, to promote health in older adults [31].

We have previously shown that adherence to guidelines for muscle strengthening activities can readily impact on sarcopenia risk in older adults [24], indicating the importance of this type of exercise activity on muscle health [35]. An important finding in our study was that changes in SRS due to reallocation of time in different PA intensities were not altered after controlling for type of PA (MSA). Thus, benefits on muscle health are promoted simply by reducing daily time being sedentary with more active pursuits irrespective of type of PA performed. However, we still believe that adherence to guidelines on PA advocating MSA with a frequency of at least two exercise sessions per week for older adults should not be overlooked given the established benefits on muscle health even in older adults who already accumulate 150 weekly minutes of MVPA [24].

Our analysis also showed that the impacts of reducing sedentary time for PA of any intensity on SRS were mirrored at the level of the muscle mass component, whereas higher intensity PA was required to infer benefits on 5-STS and HG performances. While reduction in sedentary time has been linked to beneficial effects on parameters of muscle health, discrepancies in the impact of displacing time spent in different PA intensities on components of sarcopenia risk are commonly reported [17,18]. Therefore, an analysis based on composite scores encompassing key parameters related to risk of developing sarcopenia may provide a better overall evaluation of muscle health compared to information derived from single components alone. This is particularly important in the study of muscle health in non-sarcopenic populations or for preventive purposes when the risk of sarcopenia needs to be properly evaluated.

Our study findings are strengthened by the adjustment made for adherence to protein guidelines in older adults. Indeed, adequate protein intake is an established dietary factor contributing to the maintenance of muscle mass [36], where adherence to a daily intake of 1.1 g·kg^−1^ BW has been linked to a higher muscle mass and function [26]. We further considered the potential impacts of abdominal obesity, age, medication and tobacco use. Additionally, the study outcomes further benefit from the use of objective assessment of PA behaviours. Study limitations include the cross-sectional design, which precludes inference on causality and the study sample of older adults, which is unlikely to be representative of broader populations of older adults, including different ages, ethnicities and health status. Although assessment of muscle mass may be performed with a variety of more sophisticated tools, SMI was assessed in the present study by use of bioelectrical impedance analysis, which represents a more feasible and yet valid option to capture an important dimension of sarcopenia risk, as stated by the European Working Group on Sarcopenia in Older People 2 (EWGSOP2) [1].

## 5. Conclusions

In conclusion, the present study shows that displacement of daily sedentary time with PA of at least light intensity is associated with a lower sarcopenia risk in older adults, with greater effects on SRS above the moderate PA intensity. Importantly, these findings were not influenced by the type of activities performed, emphasizing the importance of replacing sedentary behaviours with more physically active pursuits for the promotion of muscle health in older men and women.

## Figures and Tables

**Table 1:** General characteristics of the study population.

	Men	Women
*n*	88	147
Age, y	67 ± 1	67 ± 2
Body Composition		
Height, cm	178.6 ± 6.5	164.5 ± 5.6 *
Weight, kg	80.8 ± 10.7	64.8 ± 10.2 *
WC, cm	94.1 ± 9.5	80.0 ± 9.2 *
Physical Activity		
SED, min	516 ± 72	492 ± 68 *
LPA, min	274 ± 70	296 ± 66 *
MVPA, min	40 ± 21	43 ± 25
Sarcopenia Risk		
SMI, % BW	34.5 ± 3.2	26.5 ± 3.5 *
HG, kg	44.0 ± 7.1	27.7 ± 5.3 *
5-STS, s	10.2 ± 2.0	10.4 ± 2.5

Data are presented as mean ± SD. WC: waist circumference. SMI: skeletal muscle index; LPA: light physical activity; MVPA: moderate-to-vigorous physical activity; HG: Handgrip strength; 5-STS: five times sit to stand; BW: body weight. * *p* < 0.05 vs. men.

**Table 2:** Associations (ß-coefficient, 95% CI) between time spent in different physical activity intensities and SRS and its components.

	SRS	SMI	5-STS	HG
SED	0.034 [0.019 to 0.050] *	−0.044 [−0.065 to −0.023] *	−0.025 [−0.047 to −0.004] *	−0.034 [−0.055 to −0.013] *
LPA	−0.019 [−0.035 to −0.002] *	0.027 [0.006 to 0.048] *	0.015 [−0.007 to 0.036]	0.014 [−0.008 to 0.035]
MVPA	−0.101 [−0.140 to −0.062] *	0.110 [0.056 to 0.159] *	0.068 [0.015 to 0.122] *	0.127 [0.075 to 0.178] *

CI: confidence interval; SED: sedentary time; LPA: light physical activity; MVPA: moderate-to-vigorous physical activity; SMI: skeletal muscle mass index; HG: Handgrip strength; 5-STS: five times sit-to-stand. Data adjusted by accelerometer wear time. * *p* < 0.05.

**Table 3:** Effect (ß-coefficient, 95% CI) of isotemporal reallocation of time spent in different PA intensities on SRS and its subcomponents.

		SRS	SMI	5-STS	HG
Replace 10 min SED with	LPA				
	model 1	−0.019 [−0.033 to −0.006] *	0.023 [0.006 to 0.040] *	0.018 [−0.004 to 0.039]	0.017 [−0.003 to 0.037]
	model 2	−0.020 [−0.033 to −0.006] *	0.023 [0.006 to 0.040] *	-	-
	MVPA				
	model 1	−0.082 [−0.117 to −0.047] *	0.072 [0.029 to 0.115] *	0.065 [0.010 to 0.121] *	0.109 [0.058 to 0.160] *
	model 2	−0.086 [−0.120 to −0.051] *	0.075 [0.031 to 0.118] *	0.072 [0.017 to 0.127] *	0.111 [0.060 to 0.162] *
Replace 10 min LPA with	MPVA				
	model 1	−0.063 [−0.098 to −0.028] *	0.049 [0.006 to 0.092] *	0.048 [−0.007 to 0.102]	0.092 [0.042 to 0.142] *
	model 2	−0.066 [−0.101 to −0.032] *	0.051 [0.008 to 0.094] *	-	0.093 [0.043 to 0.144] *

CI: confidence interval; SED: sedentary time; LPA: light physical activity; MVPA: moderate to vigorous physical activity; SMI: skeletal muscle mass index; HG: handgrip strength; 5-STS: five times sit-to-stand. Model 1: adjusted by accelerometer wear time, waist circumference and adherence to protein intake guidelines. Model 2: model 1 + activity type (MSA). * *p* < 0.05.

## Data Availability

Data supporting reported results are available upon reasonable request with respect to ethical standards.
